# Traversing the Learning Curve Associated with a New Minimal Access Aortic Valve Replacement Service

**DOI:** 10.21470/1678-9741-2020-0436

**Published:** 2021

**Authors:** Marcus Taylor, June Low, Denish Apparau, Vipin Mehta, Rajamiyer Venkateswaran

**Affiliations:** 1 Department of Cardiothoracic Surgery, Wythenshawe Hospital, Manchester University Hospital Foundation Trust, Manchester, UK.

**Keywords:** Aortic Valve Prosthesis, Cardiac Surgical Procedures, Sternotomy, Learning Curve, Cardiopulmonary Bypass

## Abstract

**Objective:**

Isolated aortic valve replacement is a safe and frequently performed cardiac surgical procedure. Although minimal access approaches including right anterior thoracotomy and partial sternotomy have been adopted by some surgeons in recent years, concerns about additional procedural morbidity and mortality during the early phase of the learning curve persist. The aim of this study was to assess the impact of the learning curve on outcomes for a single surgeon implementing a new minimal access aortic valve replacement service.

**Methods:**

Ninety-three patients undergoing minimal access aortic valve replacement performed by a single surgeon in our institution between October 2014 and March 2019 were analysed. Patients were divided into tertiles according to procedure order. Endpoints included peri-operative mortality and post-operative complications, and these were compared across tertiles to assess the impact of the learning curve on procedural outcomes.

**Results:**

Overall in-hospital mortality was 2.15% (n=2). Despite significantly longer cardiopulmonary bypass and cross-clamp duration in the early tertile, there was no significant difference in the rate of post-operative complications, post-operative length of stay or in-hospital mortality between tertiles.

**Conclusions:**

Although our results have demonstrated a significant learning curve effect associated with the introduction of this minimally invasive approach to aortic valve replacement, as demonstrated by the significant reduction in cardiopulmonary bypass and cross-clamp duration over time, our findings suggest that a minimal access aortic valve replacement service can be safely commenced by an experienced surgeon without concerns about the learning curve significantly affecting post-operative morbidity and mortality.

**Table:** 

Abbreviations, acronyms & symbols				
ANOVA	= Analysis of variance		PS	= Partial sternotomy
AVR	= Aortic valve replacement		PVL	= Paravalvular leak
CUSUM	= Cumulative sum		RAT	= Right anterior thoracotomy
CPB	= Cardiopulmonary bypass		RIMA	= Right internal mammary artery
IQR	= Interquartile range		SD	= Standard deviation
LRTI	= Lower respiratory tract infection		SPSS	= Statistical Package for the Social Sciences
PLOS	= Post-operative length of stay		SVC	= Superior vena cava
POAF	= Post-operative atrial fibrillation		TOE	= Transoesophageal echo
PPM	= Permanent pacemaker			

## INTRODUCTION

Isolated aortic valve replacement (AVR) is one of the most commonly performed cardiac surgical procedures across the globe and is associated with low rates of operative morbidity and mortality ^[1]^. Over the last 20 years, there has been an increasing interest in undertaking this procedure via a minimal access approach, in the hope that less invasive surgery facilitates faster post-operative recovery and mobilisation, and hence a decreased rate of complications leading to an overall reduction in post-operative morbidity and mortality ^[2]^. The potential drawback associated with this technique is that the limited access can make the procedure more technically challenging, leading to an increase in cardiopulmonary bypass (CPB) and cross-clamp times ^[3]^. 

A number of different minimal access techniques for AVR have been adopted by the surgical community and are now widely accepted as safe and acceptable alternatives to surgery performed via full median sternotomy. Minimal access approaches include right anterior thoracotomy (RAT) and partial upper sternotomy (PS). Again, there are advantages and disadvantages associated with both techniques but multiple published series reviewing minimal access aortic valve replacement (mini-AVR) have demonstrated that both techniques produce outcomes at least as good as conventional aortic valve replacement ^[3,4]^. 

The aim of this study was to undertake a review of the newly established mini-AVR service in our centre to explore the learning curve associated with the introduction of this procedure and its effect on outcomes.

## METHODS

### Patient Selection

Ninety-six patients underwent mini-AVR in our institution over a 54-month period between October 2014 and March 2019 performed by a single consultant cardiac surgeon, who has been working as a consultant cardiac surgeon since 2010. Three patients who had sutureless valve implantation were excluded, as this would be a confounding variable for operative duration, leaving 93 patients included in this study. All patients scheduled to undergo isolated AVR were considered suitable for mini-AVR. The operating surgeon in this study does not currently perform concomitant atrial fibrillation (AF) surgery and hence the presence of pre-operative AF was not a contraindication to minimal access surgery. Redo procedure was not deemed to be an exclusion criterion. No isolated AVRs were performed via full sternotomy during this period. The same consultant surgeon had performed 66 isolated AVRs via full sternotomy between April 2010 and September 2014 (the 54 months immediately preceding the period of this study). 

### Data Collection

Data for all patients undergoing isolated mini-AVR were retrospectively retrieved from a digital database on a Dendrite platform. Pre-operative and peri-operative data were entered into the digital database at the time of surgery by a member of the surgical team, whilst post-operative data were entered into the database at the time of patient discharge. Categorical variables were entered using a multi-choice list and non-categorical variables were entered directly. Data missing from the digital database were sourced directly from patient case-notes. 

### Surgical Technique

After routine induction with general anaesthesia, monitoring with a central venous catheter, radial arterial line and transoesophageal echo (TOE) is established. External defibrillator pads are sited prior to skin preparation. An incision is made from just below the suprasternal notch to the level of the sternal angle. After further dissection in layers, the sternum is opened in an inverted-T fashion at the level of the 2^nd^ intercostal space. 

Arterial cannulation is done via the ascending aorta. Our technique for venous cannulation has evolved as our experience with this procedure has grown. Initially percutaneous cannulation via the right common femoral vein was favoured, with a sheath first placed in the femoral vein by the anaesthetist under ultrasound guidance. Direct right atrial cannulation with a 2-stage venous cannula was used when femoral cannulation was not possible. A number of issues with femoral cannulation, including an iliac vein injury, meant that direct right atrial cannulation was subsequently adopted as the preferred approach. However, occasional difficulty in adequately accessing the right atrial appendage for cannulation via an incision that terminates at the level of the 2^nd^ intercostal space meant that an extended incision to the level of the 3^rd^ intercostal space was sometimes necessary. To avoid this extension of the incision, direct cannulation of the superior vena cava (SVC) and insertion of a 3-stage cannula was adopted. This is now our preferred approach for venous cannulation. 

A pulmonary artery vent was initially used but it was felt that this led to overcrowding of the already limited surgical field and hence our current venting strategy is the utilisation of an aortic root vent alone.

Myocardial arrest is achieved with cold blood cardioplegia via an aortic root cannula after cross-clamping of the aorta. Direct ostial cardioplegia is used in patients with aortic regurgitation. Our technique for aortotomy, removal and replacement of the valve and subsequent closure of the aorta is no different to the technique used for conventional AVR via full sternotomy. Long-shafted needle-holders were used when placing valve sutures and a knot pusher was used to secure the sutures after valve implantation in cases in which access was particularly limited but were not used on a routine basis. Two ventricular and two atrial epicardial pacing wires are attached to the heart. Due to the manipulation of the heart required to attach the ventricular pacing wires, this part of the operation is performed prior to weaning from CPB, as is also the case for placement of the mediastinal drain, as the lack of visibility increases the possibility of ventricular injury. Weaning from CPB and closure is the same as for AVR via conventional sternotomy.

### Outcomes

The data were divided into tertiles of 31 patients according to procedure order to facilitate analysis of the learning curve associated with development of the service. It was felt that separation into three groups rather than two would allow for a more granular analysis of the learning curve and its effects on outcomes over time. 

Primary endpoints were in-hospital mortality, post-operative length of stay (PLOS) and technical failure. Technical failure was defined as one or more of the following events: peri-operative mortality (defined as a composite endpoint comprising in-hospital mortality and 30-day mortality), conversion to sternotomy, post-operative paravalvular leak, reoperation for any reason and post-operative wound infection. Secondary endpoints were need for conversion to full sternotomy, development of post-operative complications (need for blood transfusion, re-exploration, development of post-operative atrial fibrillation [POAF], need for permanent pacemaker [PPM] implantation, superficial wound infection, lower respiratory tract infection [LRTI]), readmission to critical care and post-operative paravalvular leak (PVL). 

### Statistical Analysis

Continuous variables were presented as mean±standard deviation (SD) and median±interquartile range (IQR) for normal and non-normally distributed variables, respectively. Discrete variables were presented as percentages. Distribution of continuous variables was assessed using an analysis of variance (ANOVA) test (normally distributed data) or a Kruskal-Wallis test (non-normally distributed data). Distribution of discrete variables was assessed using the chi-square test. A *P*-value of <0.05 was considered statistically significant. All statistical analysis was undertaken using SPSS version 25 (SPSS Inc, Chicago, Ill).

## RESULTS

The mean age was 71.0 years (±11.7 years) and the median logistic EuroSCORE was 5.5% (IQR 3.2-8.0%). There was no significant difference identified between the tertiles for any of the pre-operative variables analysed (Table 1). 

**Table 1:** Patient characteristics.

Variable	Group 1 (patients 1-31)	Group 2 (patients 32-62)	Group (patients 63-93)	*P*-value	Total
Age (mean±SD)	74.3 (±7.9)	69.9 (±13.2)	69.0 (±12.8)	0.163	71.0 (±11.7)
Age >75 years	45.2% (n=14)	35.5% (n=11)	35.45% (n=11)	0.665	38.7% (n=36)
Male sex	41.9% (n=13)	54.8% (n=17)	54.8% (n=17)	0.502	50.5% (n=47)
Diabetes mellitus	22.6% (n=7)	22.6% (n=7)	6.5% (n=2)	0.151	17.2% (n=16)
Hypertension	71.0% (n=22)	64.5% (n=20)	74.2% (n=23)	0.699	69.9% (n=65)
CVD	12.9% (n=4)	9.7% (n=3)	3.2% (n=1)	0.384	8.6% (n=8)
PVD	9.7% (n=3)	9.7% (n=3)	0% (n=0)	0.201	6.5% (n=6)
COPD	9.7% (n=3)	16.1% (n=5)	19.4% (n=6)	0.055	15.1% (n=14)
Redo	0% (n=0)	6.5% (n=2)	0% (n=0)	0.130	2.2% (n=2)
History of AF	19.4% (n=6)	22.6% (n=7)	6.5% (n=2)	0.188	16.1% (n=15)
NYHA ≥3	51.6% (n=16)	51.6% (n=16)	51.6% (n=16)	1	51.6% (n=48)
CKD	6.5% (n=2)	9.7% (n=3)	0% (n=0)	0.228	5.4% (n=5)
LV dysfunction*	12.9% (n=4)	12.9% (n=4)	12.9% (n=4)	1	12.9% (n=12)
Logistic EuroSCORE (median and IQR)	6.6% (IQR 4.5-9.5%)	4.8% (IQR 2.7-9.5%)	4.8% (IQR 2.5-7.2%)	0.091	5.5% (IQR 3.2-8.0%)
Urgent operation**	12.90% (n=4)	9.7% (n=3)	16.1% (n=5)	0.750	12.9% (n=12)

* Defined as left ventricular ejection fraction <50%.

** Defined as patients requiring surgery during the same hospital admission. AF=atrial fibrillation; CKD=chronic kidney disease; COPD=chronic obstructive pulmonary disease; CVD=cerebrovascular disease; IQR=interquartile range; LV=left ventricle; NYHA=New York Heart Association; PVD=peripheral vascular disease; SD=standard deviation

**Fig. 1:**
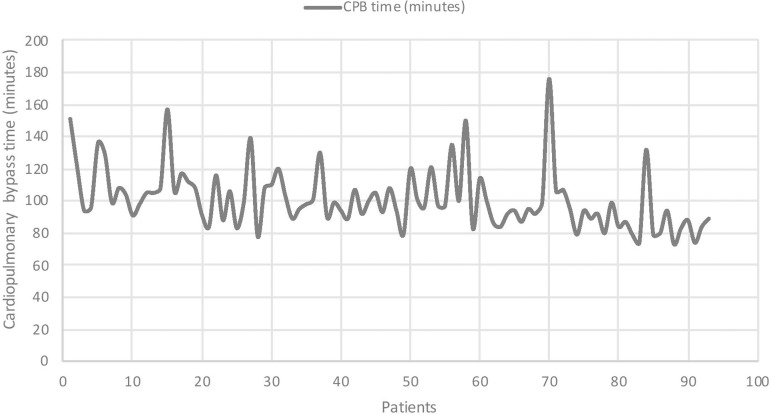
Trend of cardiopulmonary bypass times over time.

**Fig. 2:**
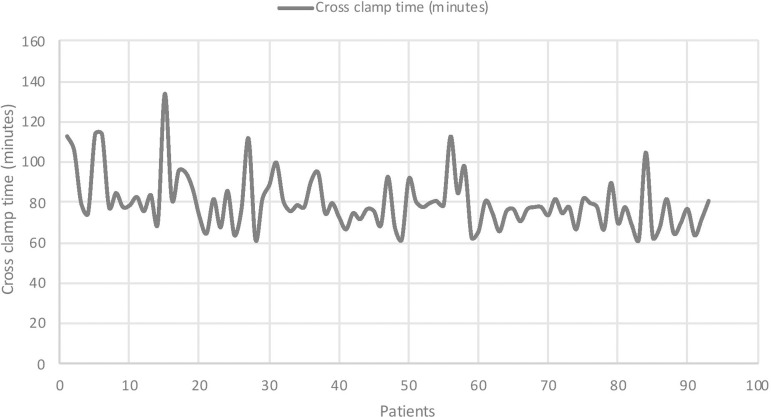
Trend of cross-clamp times over time

Intra-operative data are summarised in Table 2. The mean CPB time was 101.0 minutes (±19.0 mins) and the mean cross-clamp time was 80.3 minutes (±13.6 mins). A significant reduction in both CPB and cross-clamp duration was observed over time (*P*<0.001 for both variables). These results are shown in Figures 1 and 2, respectively. The rate of conversion to full sternotomy was 6.5% (n=6). Two of these conversions were due to poor access, two were due to abnormal TOE findings (one clot in the left ventricle and one incidental finding of a left atrial mass) and two were due to iatrogenic injury (one right ventricle puncture with Seldinger guidewire and one iliac vein injury, both occurring during femoral venous cannulation). The rate of conversion to full sternotomy was not significantly different between tertiles (*P*=0.586).

**Table 2:** Intra-operative characteristics.

Variable	Group 1 (patients 1-31)	Group 2 (patients 32-62)	Group 3 (patients 63-93)	*P*-value	Total
CPB time (mean±SD)	108.6 mins (± 18.8)	102.1 mins (± 15.5)	92.3 mins (± 19.5)	<0.001	101.0 mins (± 19.0)
Cross-clamp time (mean±SD)	86.7 mins (±17.2)	79.3 mins (±10.9)	74.9 mins (±8.8)	0.004	80.3 mins (±13.6)
Femoral vein cannulation	87.1% (n=27)	74.2% (n=23)	3.2% (n=1)	<0.001	54.8% (n=51)
Right atrial cannulation	12.9% (n=4)	25.8% (n=8)	38.7% (n=12)	0.067	21.5% (n=24)
SVC cannulation	0% (n=0)	0% (n=0)	58.1% (n=18)	<0.001	19.4% (n=18)
Conversion to full sternotomy	9.7% (n=3)	6.5% (n=2)	3.3% (n=1)	0.586	6.5% (n=6)

CPB=cardiopulmonary bypass; SD=standard deviation; SVC=superior vena cava

Overall in-hospital mortality was 2.2% (n=2). One patient was re-explored for bleeding. Two patients were found to have a mild paravalvular leak on TOE after valve implantation. Neither of these leaks were felt to be severe enough to require any additional intervention. Technical failure occurred in 12.9% (n=12) of patients. There was no significant difference identified between the tertiles for any of the post-operative variables analysed, including mortality, post-operative complications, critical care re-admission, PLOS and technical failure. These data are summarised in Table 3.

**Table 3:** Post-operative outcomes.

Variable	Group 1 (patients 1-31)	Group 2 (patients 32-62)	Group 3 (patients 63-93)	*P*-value	Total
In-hospital mortality	0% (n=0)	3.2% (n=1)	3.2% (n=1)	0.600	2.2% (n=2)
Re-exploration	0% (n=0)	0% (n=0)	3.2% (n=1)	0.364	1.1% (n=1)
Critical care re-admission	0% (n=0)	0% (n=0)	6.5% (n=2)	0.130	2.2% (n=2)
Need for blood transfusion	12.9% (n=4)	22.6% (n=7)	25.8% (n=8)	0.423	20.4% (n=19)
POAF	41.9% (n=13)	32.3% (n=10)	16.1% (n=5)	0.082	30.1% (n=28)
PPM implantation	9.7% (n=3)	0% (n=0)	6.5% (n=2)	0.228	5.4% (n=5)
Superficial wound infection	3.2% (n=1)	6.5% (n=2)	0% (n=0)	0.356	3.2% (n=3)
LRTI	6.5% (n=2)	9.7% (n=3)	9.7% (n=3)	0.872	8.6% (n=8)
Paravalvular leak	3.2% (n=1)	3.2% (n=1)	0% (n=0)	0.600	2.2% (n=2)
PLOS (median and IQR)	7 days (IQR 7-12)	6 days (IQR 5-9)	6 days (IQR 5-10)	0.102	7 days (IQR 5-10)
Technical failure	16.1% (n=5)	16.1% (n=5)	6.5% (n=2)	0.423	12.9% (n=12)

IQR=interquartile range; LRTI=lower respiratory tract infection; PLOS=post-operative length of stay; POAF=post-operative atrial fibrillation; PPM=permanent pacemaker

The 90-day and 1-year mortality were 2.2% (n=2) and 4.3% (n=4), respectively. There was no significant difference identified between the tertiles for either 90-day or 1-year mortality (*P*=0.600 and *P*=0.770, respectively). Mean follow-up time was 32 months and overall survival at the mean follow-up time point was 84.8%. 

## DISCUSSION

This study has shown that, despite significantly longer CPB and cross-clamp times during the early period of the implementation of a new minimal access aortic valve replacement service, the incidence of adverse outcomes and technical failure was not significantly higher during this early period. This demonstrates that whilst a procedural learning curve is evident, it did not affect patient safety or clinical outcomes.

Undertaking cardiac surgery via a partial sternotomy was popularised by the Cleveland Clinic during the mid-1990s and has since been adopted throughout the surgical community as a safe and effective alternative to full median sternotomy ^[5,6]^. It is now routinely performed as a first-line approach for a variety of surgical procedures in many centres across the world. Minimal access techniques have been developed in an attempt to minimise sternal disruption and hence improve the stability of the sternum after surgery, to hasten the healing process and facilitate improved cosmesis and a faster return to full activity. The minimal access approach has also been proven to significantly decrease the need for blood products ^[7]^. 

There are a number of accepted techniques for performing a mini-sternotomy. The most frequently used is the partial upper sternotomy (PS), with the horizontal sternal transection performed in either a T-shaped, V-shaped or J-shaped fashion. Parasternal, transversal, key-lock and lower hemi-sternotomy approaches have also been described ^[4,8-10]^. These different techniques represent attempts to establish an incision that maximises sternal stability and reduces post-operative lateral and craniocaudal sternal migration.

Separate to the mini sternotomy techniques are alternative sternum-sparing minimal access approaches, of which the right anterior thoracotomy (RAT), first described in the mid-1990s, is the most commonly performed ^[11]^. Although to date no high-quality randomised trials comparing RAT and PS have been undertaken, a literature review from 2017 ^[12]^ concluded that whilst post-operative length of stay (PLOS) was reduced in the RAT group (despite the significantly longer CPB and cross-clamp times), there was no overall difference in mortality between the groups. Access via a right anterolateral thoracotomy has also been described. Although more invasive than RAT, division of the right internal mammary artery (RIMA) is avoided, and if performed below the submammary line in women, it can provide excellent cosmetic results ^[13]^. 

There remains no clear consensus in the literature as to whether mini-AVR is superior to conventional AVR. A propensity-matched study using data from the United Kingdom national database comparing mini-AVR (via PS) to conventional AVR demonstrated comparable outcomes and supported mini-AVR as a safe alternative to conventional AVR ^[14]^. A recent meta-analysis comparing mini-AVR (both PS and RAT) to conventional AVR showed a reduced length of stay and reduced incidence of POAF in the mini-AVR group ^[15]^. However, the Mini-Stern trial (a UK-based randomised trial including 222 patients randomised to either PS or conventional AVR) concluded that the prolonged CPB and cross-clamp times associated with mini-AVR were not justified, as mini-AVR did not show superiority to conventional AVR in terms of mortality, complications or PLOS ^[16]^. 

Assessment of the learning curve associated with the development of minimal access techniques has also been undertaken in several other studies. Murzi et al. ^[17]^ published their experience of the first 100 patients undergoing AVR via RAT in their institution. The cumulative sum (CUSUM) analysis demonstrated no learning curve and no increased patient risk in the period immediately after adoption of the procedure. The same centre has also published an experience of the first 300 patients undergoing sutureless AVR via RAT and again demonstrated no significant learning curve for any of the six surgeons undertaking the procedures, and no difference in patient outcomes over time ^[18]^. Conversely, a review of 842 patients undergoing minimal access mitral valve repair identified that patients were significantly more likely to suffer complications at the beginning of the experience ^[19]^. These results were mirrored in a study of 3,895 patients undergoing minimally invasive mitral surgery, which demonstrated that surgeons needed to undertake between 75 and 125 operations to overcome the initial learning curve associated with the procedure ^[20]^. 

Our experience with different venous cannulation strategies throughout this series is an excellent example of how a new technique develops and is refined with both time and experience. Percutaneous femoral venous drainage (used in 54.8% of our cases [n=51]) initially seemed an attractive prospect, as it was felt that one less cannula in the small operating field would provide better access to the valve and allow a more efficient operation. However, a number of complications related to this strategy compelled us to return to traditional aorto-atrial cannulation (used in 21.5% of our cases [n=24]), before subsequently progressing to direct SVC cannulation (used in 19.4% of our cases [n=18]). Although not commonly described, there are a number of case series that describe this approach to venous cannulation in the setting of minimal access surgery ^[21,22]^. 

Despite evidence to support the adoption of minimal access approaches for AVR, some surgeons may be reluctant to adopt the technique based on concerns about a deleterious effect on outcomes, particularly during the early part of the learning curve. In our study, we have demonstrated excellent post-operative outcomes for both morbidity and mortality, even in the early phase. Interestingly, although non-significant, the median EuroSCORE was higher in the earliest tertile, which refutes the theory that outcomes in early and late groups are only comparable because of the risk-averse behaviour of selecting only extremely low-risk patients during the early phase.

Prolonged CPB and cross-clamp times are a well-described feature of minimal access cardiac surgery in comparison to traditional surgery via median sternotomy. The statistically significant differences in both CPB and cross-clamp times seen in our experience confirm that there is a learning curve associated with implementation of a new technique. However, this difference was not associated with a significant increase in the rate of either adverse outcomes (including post-operative complications, PLOS and mortality) or technical failure. Whilst the incidence of post-operative complications did not decrease over time, this is not unexpected given the relatively modest (although significant) reduction in operative times. Moreover, the aim of this study was not to show a reduction in adverse outcomes over time, but to demonstrate that the rate of complications was not significantly higher during the early stages of the implementation of the new procedure.

The single-surgeon nature of the work also raises questions about its applicability to a wider range of cardiac surgeons. The consultant cardiac surgeon in this study had been working as a consultant for almost five years prior to commencing this mini-AVR service. We feel that the outcomes demonstrated should be considered relevant to clinicians who have been working as consultant surgeons for a number of years and had little or no exposure to minimal access techniques during their training. Undertaking a similar project to assess the learning curve seen with contemporary cardiac surgery trainees who are learning minimal access aortic valve surgery as part of their training could be considered as a future project to provide additional answers to the question of the impact of the learning curve on outcomes. We also recognise that this is a single-centre and single-surgeon study and therefore the number of patients included is limited.

## CONCLUSION

Our experience demonstrates that mini-AVR is a safe and effective procedure when performed in our centre. Moreover, although our results demonstrate a reduction in operative times over time, indicating that there is a learning curve associated with this technique, the incidence of technical failure and adverse outcomes was not significantly higher in the early stage of the implementation of this procedure, suggesting that a minimal access AVR service can be safely established by an experienced surgeon.

**Table:** 

Authors' roles & responsibilities	
MT	Substantial contributions to the conception or design of the work; or the acquisition, analysis or interpretation of data for the work; drafting the work or revising it critically for important intellectual content; final approval of the version to be published
JL	Substantial contributions to the conception or design of the work; or the acquisition, analysis or interpretation of data for the work; drafting the work or revising it critically for important intellectual content; final approval of the version to be published
DA	Substantial contributions to the conception or design of the work; or the acquisition, analysis or interpretation of data for the work; final approval of the version to be published
VM	Substantial contributions to the conception or design of the work; or the acquisition, analysis or interpretation of data for the work; final approval of the version to be published
RV	Substantial contributions to the conception or design of the work; or the acquisition, analysis or interpretation of data for the work; drafting the work or revising it critically for important intellectual content; final approval of the version to be published
